# Remedies against Relapse in Intermittent Fever

**Published:** 1842-03

**Authors:** 


*Remedies against Relapse in Intermittent Fever.*—In a case of intermittent fever, which yielded readily enough to the usual remedies, but always returned when these were given up, Dr. Kuntzmann of Berlin saw the disease effectually subdued by the exhibition of a table-spoonful of the powdered stem of the pumpkin, in half a table-spoonful of tincture of wormwood. This dose was given every day at the same hour, and when indications of an approaching paroxysm were experienced. Dr. Osann says, that relapses of intermittent fever are best prevented by the use of the Tinct. Absinthii, which he has long been in the habit of prescribing, along with an equal quantity of the Tinct. Cinchon. Compos.—*Lond. and Monthly Jour. Med. Sci.* Nov. 1841, from *Hufeland's Journal*, June 1841.

